# Novel function of *Nanos2* in expression of innate immunity genes and its probable roles in maintenance of pluripotency state

**DOI:** 10.22038/ijbms.2021.53841.12104

**Published:** 2021-04

**Authors:** Monireh Bahrami, Maryam Moghaddam Matin, Moein Farshchian, Molood Asadi, Ahmad Reza Bahrami

**Affiliations:** 1 Department of Biology, Faculty of Science, Ferdowsi University of Mashhad, Mashhad, Iran; 2 Novel Diagnostics and Therapeutics Research Group, Institute of Biotechnology, Ferdowsi University of Mashhad, Mashhad, Iran; 3 Stem Cell and Regenerative Medicine Research Group, Iranian Academic Center for Education, Culture and Research (ACECR) Razavi Khorasan, Mashhad Branch, Iran; 4 Industrial Biotechnology Research Group, Institute of Biotechnology, Ferdowsi University of Mashhad, Mashhad, Iran

**Keywords:** Acetylsalicylic acid, Anti-oxidants, Epididymis, Melatonin, Sperm, Testosterone

## Abstract

**Objective(s):**

Cell-based therapeutic approaches have witnessed significant developments during the last decade especially after approval of MSCs based treatment of graft versus host disease. Several cell-based approaches have shown immunomodulatory behavior during regeneration following the unknown cascade of events but the exact mechanisms are yet to be defined. Clinical applications of cell-based drugs are hampered all over the world because of incomplete understanding of molecular mechanisms requiring the application of mechanistic approaches to solving the mystery. Current work has given us the idea that *Nanos2* enhances the cellular pluripotency characteristics while down-regulating the innate immunity genes, simultaneously.

**Materials and Methods:**

The immunomodulatory behavior of cells was studied against cells carrying the ectopic expression of *Nanos2* in comparison with *Stella* and *Oct4* individually and simultaneously using SON vector (*Stella, Nanos2* and *Oct4*).

**Results:**

It was observed that overexpression of *Nanos2* leads to down-regulation of Interferon-Stimulated Genes (ISGs)-mRNAs such as *Ifitm1*,* lsg15*, *Oas2,* and *Oas12*. *Nanos2* overexpressing MEF cells have shown restrictive inflammatory effects when cells were treated with inflammatory stimuli such as LPS and Poly (I:C).

**Conclusion:**

From our recent findings in line with many others, it can be concluded that Nanos2 acts as a coin with two sides, regulating pluripotency and immunity together which enhances resistance against inflammatory stimuli. *Nanos2* could be a potential candidate as a molecular drug for management of inflammation and immunomodulation but it requires a comprehensive comparative expression analysis of innate immunity genes *in vitro *and* in vivo*.

## Introduction

Regenerative therapies are the healing therapies based on injection, grafting, or implantation of living cells into the patient’s damaged organ or part of the body. These therapies are being developed for the ultimate cure in comparison with other medicinal approaches that are based on alleviation of the symptoms. New trends in cell-based regenerative therapies, using patient’s own tissues, have created the concept of personalized medicine (1) which has been in the clinical trials phase for a long time and a large number of disorders such as cancer (2), neurodegeneration (3), genetic (4) and cardiac disorders (5), diabetes mellitus (6), bone and joint issues (7), wounds healing (8), etc. are hoped to be treated.

Commercializing these cell-based therapies is facing several technical and ethical obstacles (9) which require more in-depth understanding of the regenerative mechanisms, especially the mechanism involved in immunomodulation and inflammation (10). The regenerative and immunomodulatory relationship has been described by several studies as a co-opt behavior of various cellular signaling pathways under the effects of simultaneous regulation of multiple tissue-specific factors as a part of understanding the regenerative mechanism from all aspects. There is a well-explained inverse relationship between regeneration and immunity in different stages of animals’ phylogenetic lineage, for example, invertebrates such as zebrafish (11), salamanders (12), etc., exhibit full regeneration capacity whereas in mammals it depends upon their development stage (13). 

A detailed knowledge of regenerative maintenance in animals, involving recognition of lost or injured tissues, and a well-mannered process of reconstruction is the pre-requisite of cell-based regenerative therapies which has not been followed in many clinical trials-based research works (14). Comprehensive research work on invertebrates has been performed to understand the regenerative mechanism and involvement of factors for body-wide patterning system modulations. Interestingly, most of these mechanisms were found to be correlated with the developmental stages of the immune system (15). Based on these studies, combinatorial approaches have been proposed to use the combination of biomaterials and cells/stem cells for achieving desired therapeutic efficacy (16, 17).

Considering the significance of regeneration maintenance as mentioned above, current work is based on the finding that *Nanos2* is a maintenance tool or a single molecular factor regulating regeneration and inflammation. In fact, it has been discussed here that *Nanos2* regulates the stemness of cells (18), following unknown immunosuppressive pathways. Current work determines the inter-relationship of innate immunity and pluripotency while proposing an idea about the underlying factors regulating innate immunity. The results might better explain how antiviral innate immunity is being regulated during differentiation of pluripotent cells.

## Materials and Methods

***Cell culture***

HEK293T cells were cultured in Dulbecco’s Modified Eagle Medium supplemented (DMEM; Thermo Fisher Science, Waltham, Massachusetts, U S A) with 10% heat-inactivated fetal bovine serum (FBS; Thermo Fisher Science, Waltham, Massachusetts, U S) and 1X penicillin-streptomycin, and then the cells were maintained in a humidified atmosphere of 5% CO_2_ at 37 °C. 

***Isolation of primary mouse embryonic fibroblasts***

Primary mouse embryonic fibroblasts (MEFs) are isolated from mouse embryos at embryonic days 13.5 to 14.5. These embryos were washed in 10 to 20 ml sterile PBS with penicillin-streptomycin. Cranial portions and viscera of each embryo were removed from the abdominal cavity using a scalpel razor and the remaining bodies were washed in fresh PBS. Digestion was performed through incubation with trypsin/ EDTA solution (Thermo Fisher Science, Waltham, Massachusetts, U S) for 30 min at 37 °C. The final cell suspension was centrifuged at 1200 *g* for 5 min. DMEM, supplemented with 10% FBS and 1% penicillin-streptomycin, was added and pipetted up and down several times to get a single cell suspension. Eventually, MEF cells were cultured in T–75 flasks and incubated at 37 °C in a humidified 5% CO_2_ atmosphere.

***Vector construction and preparation***

**Figure 1:**
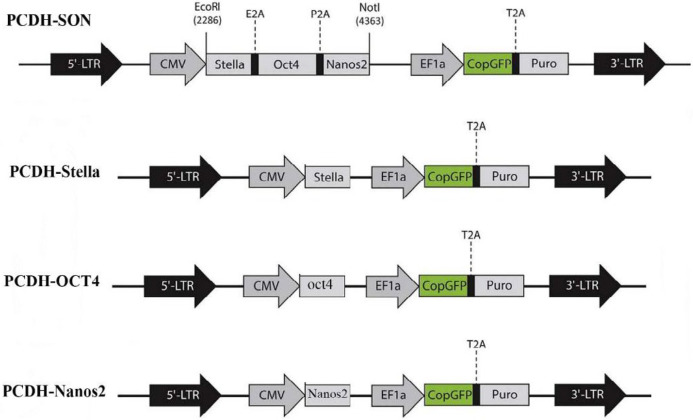
Map of the lentiviral constructs used in this study

As reported in our previous work (19), three genes: *Stella*, *Oct4*, and *Nanos2* were separately subcloned into the pCDH-513b lentiviral vector (System Biosciences, Palo Alto, CA, U S A) (Figure 1).

For this purpose, the Stella-2A-Oct4-2A-Nanos2 (SON) cassette was used as a template of PCR. Also, 3 pairs of primers were used for generation of cloning fragments, as given in Table 1.

The resulting DNA fragments from PCR and pCDH-513b lentiviral vectors were first digested with *Eco*RI and *Not*I restriction enzymes and then subjected to a ligation process using T4 DNA ligase (Thermo Scientific) according to the suggested user guide. After ligation, confirmation of the recombinant bacterial colonies was conducted with double digestion and sequencing.

***Lentiviral particle production and transduction***

HEK293T cells, at 80% confluency, were transfected with 21 μg of pCDH-SON or pCDH-Stella, pCDH-Oct4, or pCDH-Nanos2 and 21 μg of pCDH-513 (as a GFP-expressing control vector) DNA constructs, using the Trono calcium phosphate protocol. 21 μg of psPAX2 (as packaging vector) and 10 μg of pMD2G (as encoding the VSV-g) DNA constructs were used as associated vectors. Culture medium containing viral particles was collected after 48 and 72 hr, filtered through a 0.45-μm filter (Orange Scientific, Braine-l’Alleud, Belgium), and concentrated using ultracentrifugation at 28000 *g* for 1 hr at 4 ^o^C (Beckman-Coulter ultracentrifuge XL-100K, Beckman Coulter Inc, Brea, California, U.S.). 150,000 MEF cells were seeded in each well of 6-well plates and at 30%– 40% confluency; they were infected with the concentrated viral samples. 3 days after infection, the transduced MEFs were selected using Puromycin (Invitrogen, Waltham, Massachusetts, U S) Figure 1. Map of the lentiviral constructs used in this study at a concentration of 2 μg/ml. This medium was replaced by complete medium without Puromycin after 48 hr. 

***Compounds and stimulation protocols***

Lipopolysaccharide (LPS) is typically used at a concentration of 1 μg/ml for immune stimulation. In our pre-stimulation experiments, concentrations of 100 ng/ml and 1 μg/ml were tested 12 hr before collection of the cells for the gene expression analysis to every 6 well of incubated MEF cells.

For stimulation of cells with Poly (I:C) as a synthetic double-stranded RNA, Lipofectamine™ 3000 Reagent (Thermo Fisher Science, Waltham, Massachusetts, U S A) was used and the transfection procedure was followed as per manufacturer’s protocol. For this purpose, liposome-poly (I:C) complex was formed by adding 6 μl lipofectamine, 6 μl P3000™ Reagent and 1 μg/ml poly (I:C) in 150 μl serum-free DMEM (OptiMEM, Thermo Fisher Science, Waltham, Massachusetts, U S A). The whole mixture was subjected to a 20 min incubation at room temperature for proper complex formation and was then poured onto one well of the 6 wells containing 2 × 10^5^ cells cultured in 2 ml DMEM. Gene expression analysis was performed for the cell contents after 24 hr of treatment in comparison with the control samples. The same protocol was followed for MEF-pCDH-513 and MEF-pCDH-*Nanos2* cells after 10 days of transduction. 

***RNA extraction, reverse transcription, and quantitative RT-PCR***

Total RNA extraction was performed using commercial kits (Tripure, Invitrogen, Waltham, Massachusetts, U S). After DNase I (Thermo Fisher Science, Waltham, Massachusetts, U.S.) treatment, cDNA synthesis was done with M-MuLV Reverse Transcriptase (Thermo Fisher Science, Waltham, Massachusetts, U S) and quantitative RT-PCR reactions were performed with a Bio-Rad CFX-96 system (Bio-Rad, Hercules, California, U S) using the SYBR Green qRT-PCR Master Mix (Parstoos, Tehran, Iran). Gene-specific primers are given in Table 2.

## Results

***Over-expression of Stella, Oct4, and Nanos2 in MEFs***

Lentiviral expression constructs were prepared containing three genes (*Stella, Oct4, and Nanos2*) for individual and simultaneous expression. MEF cells were successfully transduced with pCDH-SON, pCDH-Stella, pCDH-Oct4, pCDH-Nanos2, and pCDH-513 constructs.

**Figure 2:**
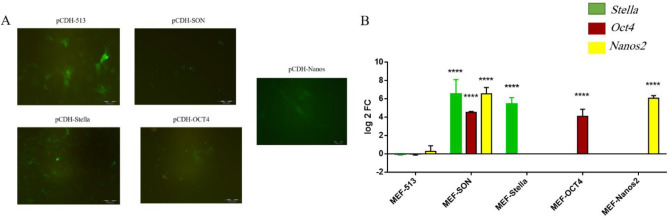
Confirmation of the transduction process for pCDH-SON, pCDH-Stella, pCDH-Oct4, pCDH-Nanos2, and pCDH-513 in the MEF cells. 2-A: Confirmation by GFP gene expression analysis using fluorescent microscopy and 2-B: Confirmation by expression of *Stella, Oct4,* and *Nanos2* genes in MEF transduced with the lentivirus

All cell populations were treated with puromycin to select the stable transformants. After 48 hr of the treatment, only cells having the gene of interest and GFP remained intact. The green color of the examined cells under the fluorescent microscopic marked the GFP gene expression, which is considered a successful transduction process (Figure 2). Real-time PCR data for every construct also showed over-expression of the target genes in the cells.

***Expression analysis of immune- and pluripotency-related genes in the engineered cells***

**Figure 3:**
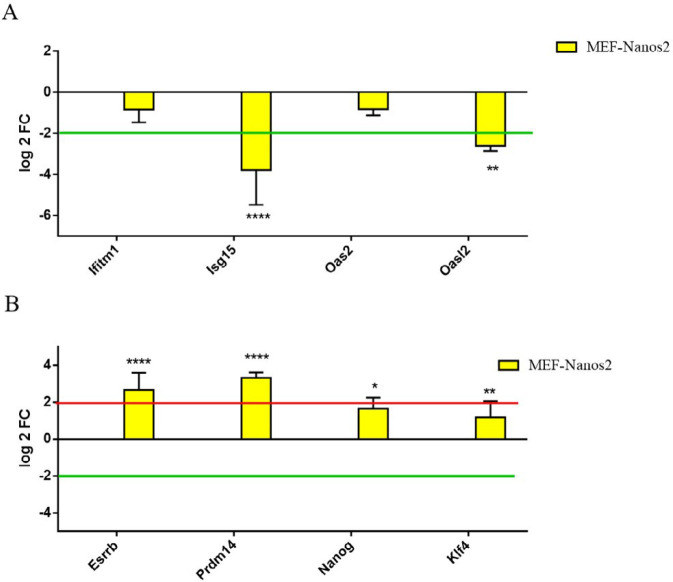
The expression of some immune-related genes (Figure 3A) and pluripotency genes (Figure 3B) in MEF, transduced with lentivirus carrying the pCDH-Nanos2 and pCDH-513 constructs as a control

Following our previous data on the negative correlation between SON gene expression and immune-related genes at the RNA level having suppressive effect of Nanos2 as shown in Figure 3A (19), here we evaluated pluripotency gene expression in MEF cells, engineered with pCDH-Nanos2 gene construct. Our results showed significant changes in some usual pluripotency and stemness markers in the engineered cells. q-RT-PCR for the quantification of *Esrrb*, *Prdm14*, and *Nanog* mRNAs showed significant over-expression (Figure 3B). 

***Expression analysis of certain immune-related genes in MEFs treated by LPS and Poly (I:C)***

**Figure 4:**
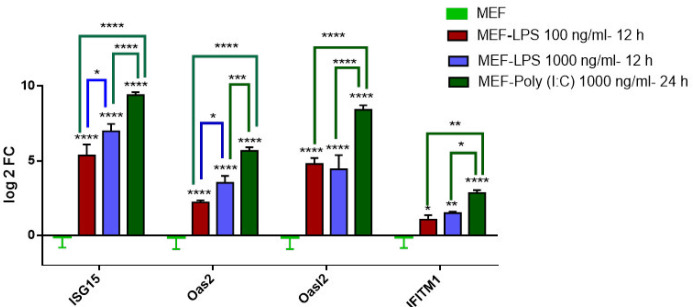
Expression of *Isg15*, *Oas2*, *Oasl2*, and *Ifitm1* genes in MEFs treated by 100 ng/ml and 1000 ng/ml LPS for 12 hr relative to the control sample (MEF). The experiment was repeated for 1000 ng/ml poly (I:C) for 24 hr too

MEFs were treated with different concentrations of LPS (100 and 1000 ng/ml) for 12 hr. RT-PCR results showed an increase in the expression of innate immunity-related genes in the LPS-induced MEF cells. The expression levels of *Isg15* and *Oas2* genes were significantly increased in MEFs, treated for 12 hr by 1000 ng/ml LPS compared with 100 ng/ml LPS (Figure 4). In the case of Poly (I:C), MEFs were treated with 1000 ng/ml for 24 hr based on previous studies, and over-expression of the selected immunity genes was obvious as shown here (Figure 4).

**Figure 5:**
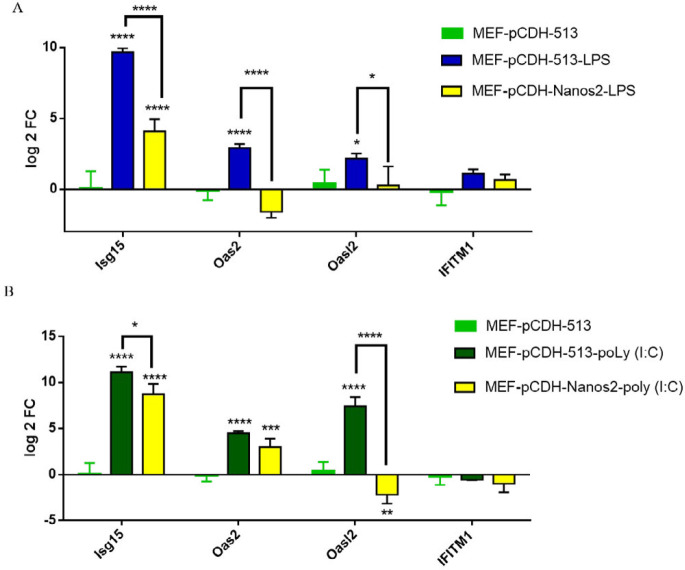
Expression of *Isg15*, *Oas2*, *Oasl2*, and *Ifitm1* genes in MEFs induced by 1000 ng/ml LPS for 12 hr (A), and 1000 ng/ml Poly (I:C) for 24 hr (B), relative to the control sample (MEF-pCDH-513)

MEFs were transducted with pCDH-513 and pCDH-Nanos2, and after 10 days of transduction, both pCDH-513 and pCDH-Nanos2 transducted MEFs were subject to stimulation of LPS (1000 ng/ml) and Poly (I:C) (1000 ng/ml) for 12 and 24 hr. Increased expression of selected innate immunity genes was observed in both Poly (I:C) and LPS treated cells, but this over-expression was significantly lower when MEFs were transducted with pCDH-Nanos2. As shown in Figure 5, pre-overexpressing *Nanos2* was followed by inflammatory stimuli such as LPS and Poly (I:C) treatment, resulting in limited induction of ISGs modulate inflammatory responses.

## Discussion

Immunomodulation in regenerative therapies is a decades-old topic of discussion among scientific communities around the globe and everyone is exploring a single part of a very big puzzle and approaches are being developed to solve this long-lasting puzzle (15). 

When trauma happens, either failure of an organ or necrotic injury inside the body, a well-defined cascade of immunomodulatory events regulates the regenerative process. These cascades are different based on the evolutionary phyletic lineage of animals, although several commonalities are found (20, 21).

Considering the relationship between immunomodulation and regeneration (14), it would be hypothesized that irrespective of the usage of stem cells or biomaterials with their unknown transformation, intrinsic factors can be activated triggering the cascade of molecular events involved in the regeneration. *Nanos2*, a well-known germ-line stem cell gene responsible for the maintenance of germ cell state (22), is also responsible for maintenance of regenerative balance through immunomodulatory properties (shown in Figure 3).

Our previous results have indicated over-expression of *Stell,* *Oct4*, and *Nanos2* individually and simultaneously suppressing the expression of ISGs like *Isg15*, *Oas2*, *Oasl2*, and *Ifitm1* genes in MEFs significantly. But *Nanos2* was found to be the most significant (19), showing an inverse relationship between immunomodulation and pluripotency (Figure 3).

A similar study has also focused on the inverse relationship between immunomodulation and pluripotency by analyzing immunity gene expression in mammalian stem cells and cells at various stages of differentiation. It was observed that ISGs expressions vary in a cell-type-specific manner, and the levels are decreased upon differentiation (23, 24). Also, recent studies have revealed that ESCs and other types of pluripotent cells do not have functional interferon (IFN)-based innate immunity (25-27). It means the IFN-based system is mainly utilized by differentiated somatic cells (28, 29) while the RNAi antiviral mechanism may be used in ESC and iPSCs (30, 31).

On the other hand, interferon immunity based on the ISGs expression has a very key role in tissue inflammation and enhanced and persistent activity of the INF signaling pathway can cause excessive inflammation and tissue damage (32, 33). Inflammatory stimuli such as LPS and Poly (I:C) stimulate the higher level of ISGs expression in MEFs when they are cultured using media with these compounds (Figure 4). However, upon ectopic expression of the cells with *the Nanos2* gene, a restrictive expression of these immune response genes was observed (Figure 5) showing some degree of resistance against immunity induction and inflammation. So the reduced response to LPS and Poly (I:C), could allow *Nanos2*-MEF to survive better under inflammatory conditions. 

In 2017, D’Angelo also demonstrated that LPS, TNF-α, and viral infection, induce robust inflammatory responses in naturally differentiated cells. But these inflammatory stimuli were unable to induce the expression of inflammatory genes in mouse embryonic stem cells, as well as human embryonic stem cells. These findings showed that embryonic stem cells are fundamentally different from differentiated somatic cells in their innate immunity and response to LPS (34). In another report, it was also observed that exposure of pluripotent stem cells to poly (I:C) and viral infection did not cause any increase in the IFN level (25, 35-38). It seems that self-renewal and pluripotency are two distinguished properties that oppose the anti-proliferative and pro-apoptotic effects of the cytokines. 

**Table 1:** List of oligonucleotide primers used for construction of the DNA fragments

Genes	Forward primer (5ʹ–3ʹ)	Reverse primer (5ʹ–3ʹ)
*Stella*	AATATTACGAATTC ATGGAGGAACCATCAGAGAAAG	AATATATCT GCGGCCGC CTAATTCTTCCCGATTTTCGC
*Oct4*	AATATTACGAATTC ATGGCTGGACACCTGGCTTCA	AATATATCT GCGG CCGC TCAGTTTGAATGCATGGGAGA
*Nanos2*	AATATTAC GAATTC ATGGACCTACCGCCCTTTGAC	AATATATCT GCGGCCGC TTATCGCTTGACTCTGCGACC

**Table 2:** Gene-specific primer sets used for quantitative RT-PCR

Genes	Forward primer (5ʹ–3ʹ)	Reverse primer (5ʹ–3ʹ)
*Nanos2*	CTGCAAGCACAATGGGGAGT	**CGTCGGTAGAGAGACTGCTG**
*Isg15*	GGTGTCCGTGACTAACTCCAT	TGGAAAGGGTAAGACCGTCCT
*Oasl2*	AGCGAGCGAGGGATGTTCAGGT	TGGGGCTGTAGGGGTTTGTCCAG
*Oas2*	GAAACTTCATTCAAACCCGGCCCA	CCGGAAGCCTTCAGCAATGTCAAA
*Ifitm1*	GTCGCTTCAGTCGTCAGCAT	TTTTCCCGTTCTTCAGCATTTGG
*RPLP*	TGGTCATCCAGCAGGTGTTCGA	ACAGACACTGGCAACATTGCGG
*Esrrb*	CTCGCCAACTCAGATTCGAT	AGAAGTGTTGCACGGCTTTG
*Prdm14*	ACAGCCAAGCAATTTGCACTAC	TTACCTGGCATTTTCATTGCTC
*Nanog*	GAACTCTCCTCCATTCTGAACCTG	GGTGCTGAGCCCTTCTGAATC
*Klf4*	GTCCTGCTCCCGTCCTTCTC	GTCGCCGCCAGGTCGTAG

Error bar indicates the mean±SD, ^****^ represents *P*-value<0.0001

Error bar indicates the mean±SD,^ *^ represents *P*-value<0.05, ^** ^represents *P*-value<0.01, *** represents *P*-value<0.001, and ^**** ^represents *P*-value<0.0001

Error bar indicates the mean±SD, ^*^ represents *P*-value<0.05, ** represents *P*-value<0.01, ^***^ represents *P*-value<0.001, and **** represents *P*-value<0.0001

Error bar indicates the mean±SD, ^* ^represents *P*-value<0.05, ^**^ represents *P*-value<0.01, ^***^ represents *P*-value<0.001, and ^**** ^represents *P*-value<0.0001

**Figure 6:**
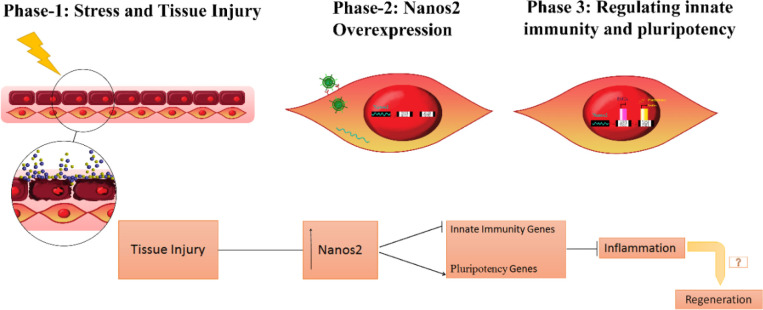
Proposed mechanism of the role of *Nanos2* in immunomodulation and inflammation management for proper regeneration of the damaged organ. Phase I: Indicates tissue injury, Phase 2 and 3 show the regenerative/surrounding cells overexpressing *Nanos2*, attenuating the ISGs expression, and inducing pluripotency genes together

## Conclusion

The concept of reducing the immunogenicity of the cells in favor of higher pluripotency and regeneration capacity has gained high momentum in the immunomodulation of stem cell research. Immune responses are critically involved in regulating the tissue repair process and must be temporally and spatially controlled for full regeneration to occur. In pro-regenerative species, it is obvious that immunity and regenerative mechanisms are finely balanced to allow proper tissue repair (39) while in pluripotent cells like the first generation of iPSCs, very reduced immunogenicity was observed (40). 

Using a combinatorial gene expression that may trigger unwanted events remained the main reason for iPSCs controversy when it comes to its clinical applications. Looking for an alternative approach to these combinations may help the scientific community to understand the secrets of regeneration and the central point of a big puzzle. *Nanos2*, an RNA binding protein which is found in germline stem cells has been associated with down-regulation of immunogenic genes (Figure 6). The current work has been performed to get a feel of the down-regulatory effect of immunity genes which could open a new chapter of discussion in cell-based therapies, demanding a highly advanced work to solve a long-lasting puzzle.
